# Histological and Radiological Evaluation of Low-Intensity Pulsed Ultrasound Versus Whole Body Vibration on Healing of Mandibular Bone Defects in Rats

**DOI:** 10.3390/medicina56090457

**Published:** 2020-09-08

**Authors:** Milad Etemadi Sh, Nan-Chen Hsieh, Seyed Shahin Movahed Mohammadi, Shahrooz Momeni, Seyed Mohammad Razavi, Javad Alizargar

**Affiliations:** 1 Department of Oral and Maxillofacial Surgery, Dental Implants Research Center, Dental Research Institute, School of Dentistry, Isfahan University of Medical Sciences, Isfahan 8415683111, Iran; Etemadi@dnt.mui.ac.ir; 2 Department of Information Management, National Taipei University of Nursing and Health Sciences, Taipei City 112, Taiwan; nchsieh@ntunhs.edu.tw; 3 Department of Oral and Maxillofacial Surgery, Faculty of Dentistry, Isfahan University of Medical Sciences, Isfahan 8415683111, Iran; 4 Department of Oral and Maxillofacial Surgery, School of Dentistry and Dental Research Center, Isfahan University of Medical Sciences, Isfahan 8415683111, Iran; drshmomeni@yahoo.com; 5 Department of Oral and Maxillofacial Pathology, School of Dentistry and Dental Implants Research Center, Isfahan University of Medical Sciences, Isfahan 8415683111, Iran; razavi@dnt.mui.ac.ir; 6 Research Center for Healthcare Industry Innovation, National Taipei University of Nursing and Health Sciences, Taipei City 112, Taiwan

**Keywords:** mandibular defects, low-intensity pulsed ultrasound, whole body vibration, bone regeneration

## Abstract

*Background and Objectives*: Mechanical stimulation can improve the structural properties of the fracture site and induce the differentiation of different cell types for bone regeneration. This study aimed to compare the effect of low-intensity pulsed ultrasound stimulation (LIPUS) versus whole body vibration (WBV) on healing of mandibular bone defects. *Materials and Methods*: A mandibular defect was created in 66 rats. The rats were randomly divided into two groups of rats. Each group was subdivided randomly by three groups (*n* = 11) as follows: (I) control group, (II) treatment with LIPUS, and (III) treatment with WBV. The radiographic changes in bone density, the ratio of lamellar bone to the entire bone volume, the ratio of the newly formed bone to the connective tissue and inflammation grade were evaluated after 1 and 2 months. *Results*: LIPUS significantly increased the radiographic bone density change compared to the control group at the first and second month postoperatively (*p* < 0.01). WBV only significantly increased the bone density compared to the control group at the second month after the surgery (*p* < 0.01). *Conclusions*: Application of LIPUS and WBV may enhance the regeneration of mandibular bone defects in rats. Although LIPUS and WBV are effective in mandibular bone healing, the effects of LIPUS are faster and greater than WBV.

## 1. Introduction

Mandibular bone defects may develop due to dental conditions, trauma, tumors, or maxillofacial surgeries, and cause complications for patients. Complete bone regeneration and regaining its normal function are ideal goals in regenerative treatments [1].

Primary bone formation is a fast process. Woven bone forms during the first weeks, which is later replaced with lamellar bone and bone marrow during the modeling and remodeling phases [2].

Several factors may affect the healing process; of which, biomechanical conditions of the fracture site are a determinant factor in this regard [3]. Mechanical stimulation can improve the structural properties of the fracture site and induce the differentiation of different cell types for bone regeneration. Thus, the fracture site may respond well to vibration during the first phases of healing [3]. Mechanical stimulation can selectively modulate osteogenesis and chondrogenesis in vivo. Vibration stimulation can induce the upregulation of cartilage-related genes such as COL2A1 and COL10A1. It also can downregulate the bone morphogenetic proteins (BMPs) [4]. Vibration is a form of mechanical stimulation that creates physical oscillation. Parameters such as range, frequency, and velocity are used to describe vibration. Whole body vibration (WBV) generates high-frequency mechanical stimulation, that is transferred to the whole body. Evidence shows that WBV affects the musculoskeletal system, improves the muscle function, increases the mineral bone density, decreases the risk of drop in muscle power, improves the muscle power, and is capable of balancing the musculoskeletal system [3]. The results of human studies on WBV have been controversial, mainly due to the use of various vibration parameters such as intensity (ranging from high intensities (3–5 g) to low intensities (<1 g)) [2]. However, high-intensity vibration is often employed by the sport clubs and is marketed in the form of exercise machines, it is not suitable for weak patients.

Low-intensity pulsed ultrasound (LIPUS) with 0.01–3 mW/cm^2^ intensity and 1.5–4 MHz frequency is a commonly used physical intervention for diagnosis and treatment. It is the only physical intervention approved by the American Food and Drug Administration to enhance healing of fresh fractures [5,6]. It seems that the mechanism of action of ultrasound is based on its thermal and non-thermal (pulsed) effects, and it can increase the circulation of blood and interstitial fluids. Non-thermal effects are more commonly seen following the application of pulsed ultrasound, and are divided into two groups: (I) Effects of cavitation as the result of expansion and contraction of gas bubbles due to pressure changes caused by ultrasound and increase in the amount of tissue fluids, and (II) unidirectional stream of fluids along the cell membranes, that causes acoustic micro-streams that affect the function and permeability of cell membranes and result in tissue regeneration. Eventually, cavitation and acoustic micro-streams activate the fibroblasts, induce protein synthesis, increase the blood flow, and enhance tissue and bone remodeling [6]. LIPUS is an effective, safe and non-invasive adjunct treatment that is being used in inducing and enhancing bone healing [7]. Studies on the efficacy of LIPUS for enhancement of mandibular defect healing are limited. Some studies reported significant effects of LIPUS on regeneration of mandibular bone defects [8] while some others reported its inefficacy in the enhancement of mandibular defects healing [9].

Moreover, to the best of the authors’ knowledge, no previous study has compared the therapeutic effects of LIPUS versus WBV for healing of mandibular defects. Thus, this study aimed to compare the efficacy of LIPUS and WBV for healing of mandibular bone defects using digital radiography and histological and histomorphometric analyses.

## 2. Materials and Methods

This animal study was conducted on 66 male Wistar rats in the Animal Research Unit of School of Dentistry, Isfahan University of Medical Sciences in 2019. The study was approved by the ethics committee of Isfahan University of Medical Sciences (IR.MUI.RESEARCH.REC.1398.608) on 26 January 2020 and conducted in accordance with the guidelines for the care and use of laboratory animals.

This study was conducted on systemically healthy, adult rats. The rats were 10 weeks old and weighed 300–350 g. The rats (*n* = 66) were randomly divided into three groups (*n* = 11) depending on the type of intervention and assessment time point (1 and 2 months): (I) control group rats with a mandibular defect, (II) rats with mandibular defects treated by LIPUS, and (III) rats with mandibular defects treated by WBV.

### 2.1. Anesthesia Induction

On the day of surgery, the rats were anesthetized according to the following protocol: For anesthesia induction, 40 mg/kg of 10% ketamine (Alfasan, Woerden, Holland) and 5 mg/kg of 2% xylazine (Alfasan, Woerden, Holland) were administered intraperitoneally. Additionally, 2% lidocaine plus 1:80,000 epinephrine was injected for local anesthesia. The mandible and hemi-cervical area were shaved, cleaned, and disinfected with 70% alcohol [10]. A circular standard-size defect with an external diameter of 5 mm, similar to the previous study of Angle et al. [11], was created in the right-side mandibular ramus. A submandibular cutaneous incision was made, and the masseter muscle was exposed. After incising the muscle along the submandibular border, a muscular flap was elevated from the buccal and lingual sides. Care was taken not to traumatize the facial nerve or the parotid duct. A through-and-through hole was drilled in the mandibular ramus using a bur with an external diameter of 5 mm (22RF050; Hagar and Meisinger, Dusseldorf, Germany). During the process of drilling, the surgical site was irrigated with saline to prevent heat injury and tissue damage. After drilling and creation of defect, the wound site was rinsed with saline and the bone chips were suctioned out [12].

Next, the wound site was sutured in all groups using simple continuous sutures with 4-0 synthetic absorbable polyglycolic acid polymer suture (Supa, Iran). The skin was sutured with 3-0 nylon simple continuous sutures (Supa, Iran).

After recovery from anesthesia, the rats were transferred to their individual cages with ad libitum access to food and water. The rats in all groups received injections of 60,000 units of penicillin (Jaber-Ebn-Hayan Pharmaceuticals, Tehran, Iran) and 5 mg of gentamycin G (Alborz Daru, Tehran, Iran) per each 1 kg of body weight intramuscularly on a daily basis for 5 days. In addition, the rats were fed 6 mg of celecoxib per each 1 kg of body weight (100 mg celecoxib; Darupakhsh, Tehran, Iran) for 5 days.

### 2.2. Treatment with LIPUS

During the first month, the rats in the LIPUS group underwent LIPUS treatment for 5 days a week, each time for 15 min. In the second month, the rats in this group underwent LIPUS treatment for 3 days a week, each time for 15 min using EXOGEN™ (Smith & Nephew Inc., Memphis, TN, USA) with the setting of 30 mW/cm^2^, 1.5 MHz, 20% duty cycle, and 1 kHz repetition rate under 1–3% anesthesia with isoflurane [11].

### 2.3. Treatment with WBV

Treatment with WBV was performed 3 days a week, each time for 20 min, and continued for 2 months with 60 Hz frequency and 1.0 mm peak-to-peak displacement. For this purpose, the rats were placed on a vibratory table in a custom-designed cage measuring 20 × 25 × 10 cm. This acrylic cage had two chambers, allowing the treatment of two rats at a time while they were physically separated from each other [13].

### 2.4. Digital Radiography

Digital radiographs were obtained at 0, 1 and 2 months [12], and bone healing was evaluated according to the change in the gray scale value in the time periods of 0–1 month and 0–2 months. All radiographs were obtained using a digital X-ray unit (Cygnus Technologies, Scottsdale, USA) with a charge-coupled device sensor (CCD, 27.38 mm) size 2 and Dr. Suni software (Suni Medical Imaging, San Joe, USA). The X-ray sensor was adapted to the jawbone as much as possible, and the X-ray was irradiated parallel between the X-ray sensor and jawbone. As a calibration control for each image, a 2-mm orthodontic wire has been fixed on the sensor surface. All X-rays were taken under anesthesia.

### 2.5. Histopathological Assessment

Histopathological assessment was performed at 1 and 2 months, postoperatively. For this purpose, the rats were sacrificed by high-dose chloroform. Specimens were evaluated radiographically at this moment. The specimens were fixed in 10% formalin for 24 h and rinsed for 4 h after that. For assessment of decalcified tissues, all specimens of each group were decalcified in 10% formic acid, dehydrated with increasing concentrations of ethyl alcohol and were embedded in paraffin. Each specimen was sectioned into 6 µm slices longitudinally. The tissue sections were immersed in 65 °C hematoxylin for 5 min and were then rinsed with water and differentiated by acid-alcohol. For the next stage, they were immersed in eosin dye and rinsed with ascending concentrations of alcohol [11]. They were then immersed in xylol and mounted on slides. Each slide was coded for the purpose of blinding in all phases of the study (treatment, histological sampling, and pathological assessment). After that, the specimens were inspected under a light microscope (OLYMPUS CXZFIS, Tokyo, Japan). Type of bone (woven or lamellar), presence of connective tissue, and type of inflammation (acute, chronic, or mixed), and inflammation grade as no inflammation, low inflammation (lower than 30%), intermediate (30% to 60%) and sever (more than 60%) were determined in this phase.

### 2.6. Histomorphometric Assessment

For histomorphometric assessment, the stained tissue specimens were inspected under the same light microscope at ×40 and ×100 magnifications. The percentage of woven and lamellar bone, as well as the percentage of connective tissue in the defects, was calculated using a graded lens at ×100 magnification [14]. Using Photoshop software, the number of pixels of the entire image was first calculated and then the percentage of newly formed bone was calculated according to the ratio of bone pixels to the entire image pixels. The results of each group have been measured and been averaged to stand for each group value.

### 2.7. Statistical Analysis

All analyses were performed using SAS vers. 9.4 (SAS, Cary, NC, USA) at a significance level of *p* < 0.05. Shapiro–Wilk test was used to test the normality. One-way analysis of variance (ANOVA) and descriptive analysis were used and boxplots showed the difference between the statistically significant results. After the results had been calculated, the power of the study was determined by using a free online open source calculator (Georgia version 3, OpenEpi, Atlanta, GA, USA). The method of calculating power was “comparing two means” for the statistically significant variable(s) for the interventional and control groups at the first and second month separately (https://www.openepi.com/Power/PowerMean.htm).

## 3. Results

Seven rats died during the experiment. Bleeding did not occur, and blood clot did not form at the surgical site in any rat. The surgical skin incisions completely healed by day 7 postoperatively, and all sutures were removed.

**Figure 1:**
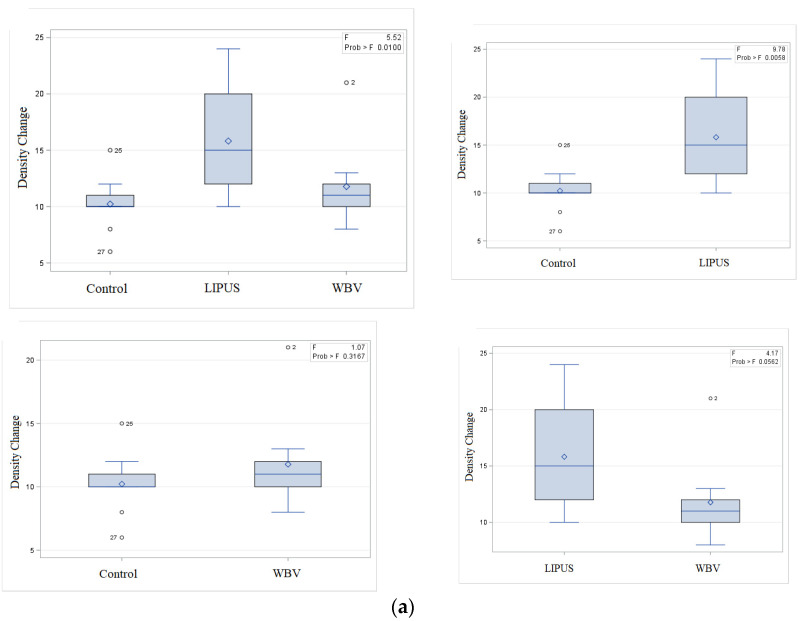

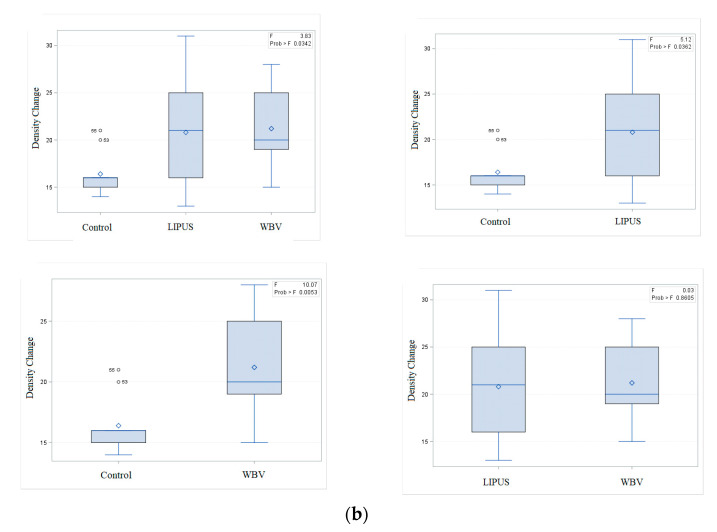
Boxplot distribution of density change between the three groups (**a**) After one month (**b**) After two months.

**Figure 2:**
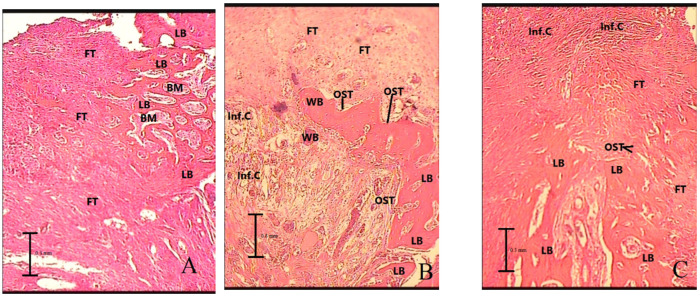
Histologic findings of mandibular bone defect in groups 1 month after surgery with magnification of ×100. (**A**) Control, (**B**) low-intensity pulsed ultrasound (the mandibular bone defect was mainly replaced by lamellar bone), (**C**) whole-body vibration (the mandibular bone defect was mainly replaced by lamellar and woven bone). LB: lamellar bone, WB: woven bone, FT: fibrosis or connective tissue, Inf.C: inflammatory cells, BM: bone marrow, OST: osteoblast.

**Figure 3:**
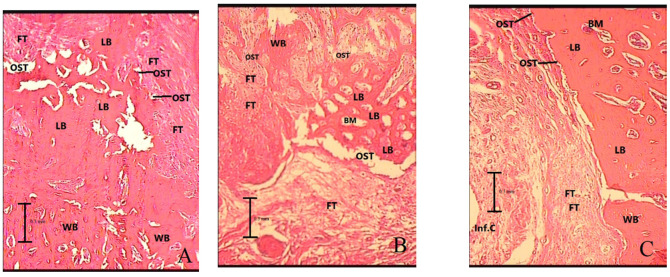
Histologic findings of mandibular bone defect in groups 2 months after surgery with magnification of ×100. (**A**) Control (the mandibular bone defect was mainly replaced by lamellar bone), (**B**) low-intensity pulsed ultrasound (the mandibular bone defect was mainly replaced by lamellar and woven bone), (**C**) whole-body vibration (the mandibular bone defect was mainly replaced by lamellar bone). LB: lamellar bone, WB: woven bone, FT: fibrosis or connective tissue, Inf.C: inflammatory cells, BM: bone marrow, OST: osteoblast.

Table 1 and Table 2 present the mean (± standard deviation) inflammation grade, change in bone density, the percentage ratio of the newly formed bone to the connective tissue and the percentage ratio of the lamellar bone at 1 and 2 months after the surgery. Figure 1 shows the boxplots of distribution of bone density change in the experimental groups and the histologic findings of the mandibular bone defect region in three groups are shown in Figure 2 and Figure 3.

Radiographic bone density change was statistically higher in the LIPUS group compared to the control group at the first month. This index was also higher in WBV and LIPUS compared to the control group at the second month. WBV group had a higher grade of inflammation at the second month compared to the LIPUS group.

The power of the study was calculated for LIPUS and control groups at the first month, and the LIPUS and control group at the second month and WBV group and control group at the second month were 91.39%, 61.95% and 88.75%, respectively.

## 4. Discussion

In this study, histopathological and histomorphometric evaluation showed no significant difference in bone regeneration between LIPUS and WBV compared to the control group. However, as proven by the radiographic evaluation, WBV and LIPUS can enhance the bone healing and the effects of LIPUS can be faster than WBV. Evidence supporting the efficacy of LIPUS and WBV for regeneration of maxillofacial bone defects and bone healing is inconclusive, and it is not known whether these modalities can enhance the regeneration of bone defects and increase the bone density in this area.

Although evidence shows that LIPUS can potentially induce bone regeneration in long bones, its therapeutic effect on maxillofacial bone has not been well elucidated, which may be due to different fetal entities of long bones and maxillofacial bones. The ossification of maxillofacial bones occurs via the intramembranous ossification while the ossification process of long bones is endochondral [9,15].

The results of this study obtained at 1 month revealed that the application of LIPUS increased the bone density compared with the control group. No significant difference was noted between the WBV group with the LIPUS (*p* value = 0.056) or the control (*p* value = 0.316) group. The process of bone healing has three phases. The first phase includes coagulation and inflammation, and occurs in the first week, postoperatively. Angiogenesis and proliferation occur in the second phase. Evidence shows that LIPUS may have the highest efficacy in this phase [16,17] Finally, woven bone forms following the formation of primary connective tissue. This phase is an important step in the intramembranous ossification of maxillofacial bones. LIPUS may also be effective in this phase [18].

LIPUS generates a type of mechanical energy that is transferred in the form of acoustic pressure waves through the tissues. It is absorbed by the tissues depending on their density. The micromechanical variants generated by the pressure waves in the human tissue can lead to biochemical events at the cellular level, and enhance the healing process. Osteoblasts are sensitive to mechanical loads. Thus, LIPUS can directly stimulate the proliferation of osteoblasts mechanically, induce the ossification of cartilage, and increase bone mineralization. Other mechanisms of action of LIPUS include change in function of cell membrane and subsequently increased the level of cellular calcium, stimulation of fibroblast activity, increased protein synthesis, enhanced permeability of vessels and angiogenesis [19]. It seems that LIPUS increases the expression of genes involved in osteogenesis by osteoblasts such as Runt-related transcription factor-2, alkaline phosphatase, osteorix, and cyclin-D1, which explains the pro-osteogenesis effects of LIPUS [20].

Bronoosh et al. [9] evaluated the effects of LIPUS with 3 MHz frequency on the regeneration of mandibular defects in rabbits, and reported that the amount of newly formed bone in the LIPUS group was significantly greater than that in the control group after 4 weeks, which was in agreement with our findings; although the frequency of LIPUS in their study was different from ours. El-Bialy et al. [21] also showed the positive effects of LIPUS on mandibular bone regeneration in rats which underwent distraction osteogenesis. Erdogan et al. [15] demonstrated an increase in the bone volume following the application of LIPUS with 1.5 MHZ frequency for mandibular fracture healing in rabbits.

Angle et al. [11] reported that application of LIPUS on femoral bone defects in rats did not enhance healing. They concluded that LIPUS is not osteo-inductive but can increase osteogenic differentiation. However, in their study they tested the synergic effect of recombinant human bone morphogenetic protein-2 (rhBMP-2) with LIPUS on bone formation, so the sole effect of LIPUS cannot be fairly discussed. Schortinghuis et al. [12] discussed that application of LIPUS did not induce bone regeneration in large mandibular bone defects in rats. Their results were different from ours, which may be due to the fact that they used a hand-made LIPUS device, so make their results incomparable to our findings. The clinical protocol for the application of LIPUS includes the application of 1 MHZ sine waves with 1 KHz frequency, a mean intensity of 30 mW/cm^2^ and pulse width of 200 ms for 20 min a day [19]. However, no consensus has been reached regarding the frequency of LIPUS for therapeutic purposes.

A study by Ruppert et al. [22] conducted a rodent study in a femoral intramedullary implant model. They had 4 groups: control, locally applied vibration, LIPUS, and combined treatment to determine the effects of those treatments on healing 4-week and 8-week postoperatively. The main results of their study showed that LIPUS had better results compared to the locally applied vibration for accelerating osseointegration and increasing bone-implant failure loads, although the vibration also increased the bone around the implants. Their results also showed that the LIPUS benefits on osseointegration at 4 weeks were not maintained at 8 weeks. Although this study settings were different from our study, it confirms the superiority of LIPUS on vibration in osteogenesis.

The results of this study at 2 months after the surgery revealed that the application of WBV increased the bone density compared with the control group, which may indicate the osteogenic effects of WBV on bone tissue [13]. The vibration treatment is a low-impact exercise. The mechanical signals are received by the osteocytes as mechanical load, leading to bone formation and prevention of bone resorption [13]. As explained by Rubin et al. [23] the bone tissue becomes stronger when subjected to mechanical loads. The cells that are responsible for the production and maintenance of tissues in the musculoskeletal system are derived from the bone marrow progenitor cells, these stem cells are capable of differentiating into several cell lines such as osteoblasts, which are involved in the bone formation process. It has been confirmed that mechanical stimuli can induce the differentiation of stem cells to osteogenic lines via the mechano-transduction mechanism [13]. Matsumoto et al. [24] reported that the application of low-intensity WBV enhance the regeneration of bone defects in rat tibia, and correlates with the vascular modulation. Chen et al. [18] performed a systematic review on the efficacy of WBV for fracture healing in ovariectomized rats and reported an increase in the total bone volume after 8 weeks. Butezloff et al. [13] reported that application of WBV can increase the bone mineral density in ovariectomized rats. Their results were in accordance with our findings.

Our study had certain limitations: different frequencies of vibration could be induced and be compared to other studies. Larger sample sizes could also increase the power of the study, especially regarding WBV. The other fact that should be considered by the researchers in this field is that by only measuring the bone density we cannot clearly evaluate all the parameters involved in the effects of WBV on bone regeneration, so studies that test other parameters as well as bone density are needed to confirm our results. These studies are required to confirm the current findings and assess the molecular and cellular mechanisms involved in the efficacy of LIPUS and WBV in the maxillofacial region.

## 5. Conclusions

Application of LIPUS with 1.5 MHz frequency for 1 month and application of WBV for 2 months postoperatively may enhance osteogenesis and bone healing in rats; however, the difference between the efficacy of LIPUS and WBV for osteogenesis is not prominent.

## Figures and Tables

**Table 1:** Distribution of bone healing parameters in the interventional and control group at 1 month after surgery.

Parameter	Groups			Between 3 Groups *p* Value	WBV-Control *p* Value	LIPUS-Control *p* Value	WBV-LIPUS *p* Value
	WBV (*n* = 9)	LIPUS (*n* = 11)	Control (*n* = 9)				
Lamellar Bone Ratio	53.111	52.455	51.111	0.296	0.204	0.194	0.593
	±3.586	±1.650	±2.770				
Bone Connective Ratio	1.033	1.062	1.023	0.914	0.933	0.503	0.797
	±0.334	±0.131	±0.116				
Radiographic Bone Density Change	11.778	15.818	10.222	0.010	0.316	0.005	0.056
	±3.767	±4.854	±2.488				
Inflammation Grade	2.11	1.63	1.77	0.500	0.344	0.726	0.322

*p*-value based on one-way ANOVA test; data of the groups presented as mean ± SD, SD: standard deviation; WBV: whole body vibration; LIPUS: low-intensity pulsed ultrasound.

**Table 2:** Distribution of bone healing parameters in the interventional and control group at 2 months after surgery.

Parameter (Mean ± SD)	Group			Between 3 groups *p* Value	WBV-Control *p* Value	LIPUS-Control *p* Value	WBV-LIPUS *p* Value
	WBV (*n* = 10)	LIPUS (*n* = 10)	Control (*n* = 10)				
Lamellar Bone Ratio	53.500	53.650	52.550	0.724	0.596	0.181	0.929
	±5.142	±1.270	±2.153				
Bone Connective Ratio	1.156	1.259	1.112	0.231	0.567	0.084	0.315
	±0.214	±0.231	±0.105				
Radiographic Bone Density Change	21.200	20.800	16.400	0.034	0.005	0.036	0.860
	±4.211	±5.711	±2.270				
Inflammation Grade	2.60	1.80	2.50	0.060	0.722	0.093	0.041

*p*-value based on one-way ANOVA test; data of the groups presented as mean±SD, SD: standard deviation; WBV: whole body vibration; LIPUS: low-intensity pulsed ultrasound.

## References

[1] Lynch S.E., Marx R.E., Wisner-lynch L.A. (2008). Tissue Engineering: Applications in Maxillofacial Surgery and Periodontitis.

[2] Thompson W.R., Yen S.S., Rubin J. (2014). Vibration therapy: Clinical applications in bone. Curr. Opin. Endocrinol. Diabetes Obes..

[3] Wang J., Leung K.S., Chow S.K., Cheung W.H. (2017). The effect of whole body vibration on fracture healing—A systematic review. Eur. Cell Mater..

[4] Hudolin T., Kastelan Z., El-Saleh A., Bakula M., Coric M., Kes P., Tomas D., Basic-Jukic N. (2020). Bone morphogenic proteins-2, -4, -6 and 7 in non-muscle invasive bladder cancer. Oncol. Lett..

[5] Lou S., Lv H., Li Z., Tang P., Wang Y. (2018). Effect of low-intensity pulsed ultrasound on distraction osteogenesis: A systematic review and meta-analysis of randomized controlled trials. J. Orthop. Surg. Res..

[6] Tehranchi A., Badiee M., Younessian F., Badiei M., Haddadpour S. (2017). Effect of Low-intensity Pulsed Ultrasound on Postorthognathic Surgery Healing Process. Ann. Maxillofac. Surg..

[7] Lou S., Lv H., Li Z., Zhang L., Tang P. (2017). The effects of low-intensity pulsed ultrasound on fresh fracture: A meta-analysis. Medicine.

[8] Patel K., Kumar S., Kathiriya N., Madan S., Shah A., Venkataraghavan K., Jani M. (2015). An Evaluation of the Effect of Therapeutic Ultrasound on Healing of Mandibular Fracture. Craniomaxillofac Trauma Reconstr..

[9] Bronoosh P., Tanideh N., Noorafshan A., Andisheh Tadbir A., Aalipanah M., Kamali F., Abbasnia K., Koohi-Hosseinabadi O. (2015). Effects of low-intensity pulsed ultrasound on healing of mandibular bone defects: An experimental study in rabbits. Int. J. Oral Maxillofac. Surg..

[10] Kaban L.B., Glowacki J. (1981). Induced osteogenesis in the repair of experimental mandibular defects in rats. J. Dent. Res..

[11] Angle S.R., Sena K., Sumner D.R., Virkus W.W., Virdi A.S. (2014). Combined use of low-intensity pulsed ultrasound and rhBMP-2 to enhance bone formation in a rat model of critical size defect. J. Orthop. Trauma.

[12] Schortinghuis J., Ruben J.L., Raghoebar G.M., Stegenga B. (2004). Ultrasound to stimulate mandibular bone defect healing: A placebo-controlled single-blind study in rats. J. Oral Maxillofac. Surg..

[13] Butezloff M.M., Zamarioli A., Leoni G.B., Sousa-Neto M.D., Volpon J.B. (2015). Whole-body vibration improves fracture healing and bone quality in rats with ovariectomy-induced osteoporosis. Acta Cirt. Bras..

[14] Gerstenfeld L.C., Wronski T.J., Hollinger J.O., Einhorn T.A. (2005). Application of histomorphometric methods to the study of bone repair. J. Bone Miner. Res..

[15] Erdogan O., Esen E., Ustun Y., Kurkcu M., Akova T., Gonlusen G., Uysal H., Cevlik F. (2006). Effects of low-intensity pulsed ultrasound on healing of mandibular fractures: An experimental study in rabbits. J. Oral Maxillofac. Surg..

[16] Azuma Y., Ito M., Harada Y., Takagi H., Ohta T., Jingushi S. (2001). Low-intensity pulsed ultrasound accelerates rat femoral fracture healing by acting on the various cellular reactions in the fracture callus. J. Bone Miner. Res..

[17] Spadaro J.A., Albanese S.A. (1998). Application of low-intensity ultrasound to growing bone in rats. Ultrasound Med. Biol..

[18] Chen J., Ruan H., Liu Y., Bao J., Xu H., Yao M., Cui X., Liang Q., Wang Y. (2018). Therapeutic effects of whole-body vibration on fracture healing in ovariectomized rats: A systematic review and meta-analysis. Menopause.

[19] Ezzati Givi M., Pey S. (2016). Evaluation of Low Intensity Pulsed Ultrasound Effects on the Osteogenesis Potential of Demineralized Bone Matrix in Experimental Tibial Defect in Rabbits. Jentashapir J. Cell Mol. Biol..

[20] Zhou X.Y., Wu S.Y., Zhang Z.C., Wang F., Yang Y.L., Li M., Wei X.Z. (2017). Low-intensity pulsed ultrasound promotes endothelial cell-mediated osteogenesis in a conditioned medium coculture system with osteoblasts. Medicine.

[21] El-Bialy T.H., Royston T.J., Magin R.L., Evans C.A., Zaki Ael M., Frizzell L.A. (2002). The effect of pulsed ultrasound on mandibular distraction. Ann. Biomed. Eng..

[22] Ruppert D.S., Harrysson O.L.A., Marcellin-Little D.J., Bollenbecker S., Weinhold P.S. (2019). Osteogenic benefits of low-intensity pulsed ultrasound and vibration in a rodent osseointegration model. J. Musculoskelet. Neuronal Interact..

[23] Rubin C.T., Sommerfeldt D.W., Judex S., Qin Y.-X. (2001). Inhibition of osteopenia by low magnitude, high-frequency mechanical stimuli. Drug Discov..

[24] Matsumoto T., Goto D. (2017). Effect of low-intensity whole-body vibration on bone defect repair and associated vascularization in mice. Med. Biol. Eng. Comput..

